# Achieving the Millennium Development Goals: Does Mental Health Play a Role?

**DOI:** 10.1371/journal.pmed.0020291

**Published:** 2005-09-13

**Authors:** J. Jaime Miranda, Vikram Patel

## Abstract

Miranda and Patel argue that mental disorders are among the most important causes of disability and premature mortality in developing countries.

The Millennium Development Goals (MDGs) have captured the attention of the international health and development community in recent years [1–6], and in 2003 two world reports—the Human Development Report and the World Health Report—concentrated specifically on these goals [7,8]. The MDGs provide a vision for development in which health and education are squarely at the centre [1,9]. Three of the eight goals, eight of the 16 targets, and 18 of the 48 indicators relate directly to health [9,10]. Health is also an important contributor to several other goals.

Intriguingly, the health goals almost entirely ignore noncommunicable diseases, including mental disorders. Yet there is compelling evidence that in developing countries mental disorders are amongst the most important causes of sickness, disability, and, in certain age groups, premature mortality. Mental health–related conditions, including depressive and anxiety disorders, alcohol and drug abuse, and schizophrenia, contribute to a significant proportion of disability-adjusted life years (DALYs) and years lived with disability (YLDs), even in poor countries [11]. Apart from causing suffering, mental illness is closely associated with social determinants, notably poverty and gender disadvantage, and with poor physical health, including having HIV/AIDS and poor maternal and child health. Yet mental health remains a largely ignored issue in global health, and its complete absence from the MDGs reinforces the position that mental health has little role to play in major development-related health agendas.

This article seeks to question this assumption. Using evidence on mental health in developing countries, we argue that addressing mental health problems is an integral part of health system interventions aimed at achieving some of the key MDGs. [Fig pmed-0020291-g001]

**Figure pmed-0020291-g001:**
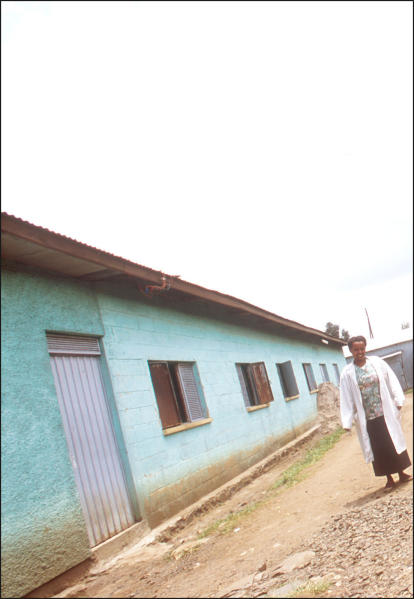
Ammanuel Psychiatric Hospital in Addis Ababa, Ethiopia (Photo: © WHO/P.Virot)

## Mental Health and the MDGs

Below, we consider the evidence linking mental health with three MDGs: eradicating poverty, reducing child mortality, and improving maternal health. However, the relevance of mental health is not limited to these goals alone. For example, a major reason why children are not able to either enrol in schools or complete primary education (MDG 2) is related to developmental and mental disorders, for example, learning disabilities [12]. There are several areas of confluence between HIV/AIDS (MDG 6) and mental health [13]—for example, people with HIV/AIDS are much more likely to suffer mental health problems, and these problems in turn can affect their overall health outcomes.

### Goal 1: Eradicate extreme poverty and hunger

Stressful life experiences such as exposure to violence and poor physical health, which are well-recognized risk factors for mental disorders, are more likely to be experienced by poor people. Thus, it is not surprising that virtually all population-based studies of the risk factors for mental disorders, particularly depressive and anxiety disorders, consistently show that poor and marginalized people are at greater risk of suffering from these [14]. We also know that mental disorders impoverish people because of both increased costs of health care—often being sought through private providers—and lost employment opportunities. Most mental illnesses are relatively simple, and cheap, to treat, and evidence from clinical trials shows that efficacious treatment is associated with significant reductions in overall health-care costs [15]. Thus, treating mental disorders, particularly in the poor, who bear a disproportionate burden of suffering, would help people with mental disorders work more productively and reduce their health-care expenditures, facilitating the conditions necessary to rise out of poverty.

### Goal 4: Reduce child mortality

A series of studies from South Asia have shown that early childhood failure to thrive, as indicated by undernutrition and stunting in babies under a year old, is independently associated with depression in mothers [16]. For example, a recent population-based cohort study from Pakistan has shown that babies of mothers who were depressed during pregnancy and in the postnatal period had a risk more than five times greater of being underweight and stunted at six months than babies of nondepressed mothers, even after adjustment for other known confounders such as maternal socioeconomic status [17]. Childhood failure to thrive is a major risk factor for child mortality; thus, it would be plausible to hypothesize that depression in mothers is also associated with increased child mortality. Indeed, evidence shows that depressed mothers are more likely to cease breast-feeding, and that their babies are significantly more likely to suffer diarrhoeal episodes or to not have their complete immunizations [17], all of these being recognized risk factors for childhood mortality. This study also showed that depression during pregnancy was strongly associated with low birth weight, an association that has been replicated in studies in India [18] and Brazil (S. Mitsuhiro, C. Ferri, V. Patel, M. Barros, E. Chalen, et al., unpublished data).

### Goal 5: Improve maternal health

One of the most common health problems affecting mothers during pregnancy and after childbirth is depression. A large number of studies from most regions of the developing world show that 10%–30% of mothers will suffer from depression [19–21]. This condition is typically missed, not least because many of its core features such as fatigue and poor sleep are also commonly associated with motherhood itself. However, it is no trivial condition. Apart from its effect on the child, as described above, there is evidence that maternal depression can profoundly affect mothers themselves. Depressed mothers are much more disabled and less likely to care for their own needs. Suicide is a leading cause of maternal death in developed countries [22]. Suicide is now a leading cause of death in young women in the reproductive age group in the world's two most populous countries, India and China [23,24]. It is plausible that depression in mothers may also lead to increased maternal mortality, both through adversely affecting physical health needs as well as more directly through suicide.

## Challenges to Acknowledging Mental Health in the MDGs

There are five major challenges to acknowledging mental health in the MDGs. The first, and perhaps the greatest, challenge lies in the very nature of the MDGs themselves. Although the MDGs have been portrayed as a consensus view of international development, it has been questioned whether it is worthwhile to have ambitious goals of this nature, given the patchy record of implementation of previous international declarations [1]. Examples of this patchy record include the failure of the international community to respect and fulfil the values expressed in the *Universal Declaration of Human Rights* [25], the failure to achieve the goal of the *Declaration of Alma-Ata* [26], and the failure to meet the international targets for sexual and reproductive health promoted during the last decade [27].

Second, national ownership of the goals is an important issue [1,8]. The power and purpose of the MDGs is that they are supposed to represent a means by which people can hold authorities accountable. There is a risk, however, that the MDGs are seen by some developing countries as being of primary concern to donors; they may be perceived as a new form of conditionality and as too restrictive in their scope to cover the multifaceted nature of health and development. In countries that have already achieved some of the MDGs, such as many countries in Latin America and Asia where targets for child mortality and maternal mortality are already met, the one-size-fits-all prescription suggested by the MDGs may not have local validity. Even though communicable diseases remain virtually the sole priority for global health policy, they do not constitute the major contributor to burden of disease in any region of the world apart from sub-Saharan Africa [28]. Even as mental health is now being prioritised as a major health problem in several developing countries, ironically their concerns do not find a place in global health targets and agendas.

Third, if mental health has a role to play towards meeting the MDG targets and health development goals, its role is likely to be more evident at the local health service level than at the level of international discourse. Nevertheless, the MDGs do not address strengthening of health systems [1,2,5,29,30]. This failure to address health systems raises important concerns because it risks diverting resources in under-resourced and overstretched services towards activities aimed at achieving specific targets. As a consequence, mental health and a host of other health problems, particularly those of a chronic and noncommunicable nature—which require a strong health system to deliver effective, multicomponent interventions—fall further by the wayside.

Fourth, as in any other plan with specific targets, the ultimate aim is to achieve a particular set of indicators that are expressed as national averages, but these averages may end up masking ongoing inequities. Significant progress in groups other than the poor can, for example, result in the achievement of the targets, with only minor improvements in the health status of the poor [1,31]. In terms of mental health, it is necessary to be conscious about this limitation because it is known that the least advantaged groups in society are the ones that carry the greater burden of mental illness. The stigma associated with mental illness that already serves to hide the suffering of countless millions is further compounded by being altogether ignored in the new programs focused on achieving the MDG targets.

Finally, the MDGs have been outlined with a specific number of objectives, targets, and indicators, which serve as standards for comparability purposes. Unfortunately, none of the targets or indicators devised for the MDGs [10] have a specific connection with mental health, nor do they enable development of monitoring methods that address mental health.

## Implications for Global Policy

It is surprising that, while the developed world is investing substantial funds into mental health care and mental health promotion programs for its own populations, the leaders of the MDG project, international donors, and multilateral agencies, all of which are heavily represented by the developed world, have chosen to completely ignore mental health in the agenda for the health of the developing world. They have chosen this course of action, despite evidence of the burden of mental disorders, their association with the MDGs, and, perhaps most importantly, evidence that they can be effectively treated using locally available and affordable resources [32].

Are poor people in developing countries less deserving of mental health care? It is commonly argued that poor people in developing countries have more serious physical health problems to contend with and, therefore, the scarce resources that are available should be allocated to such problems. However, the evidence, some of which we have briefly outlined above, clearly shows that mental health has an integral role to play in achieving many of the MDGs. Can we provide effective health care for mothers or people living with HIV/AIDS, for example, without addressing their mental health needs?

Our prescription for global policy is to urge those involved with implementing and funding programs aimed at achieving the MDGs to take a broad and holistic approach to the targets. This approach would imply an explicit focus on strengthening basic health-care systems, for example, by strengthening the availability and skills of health workers, not only to deliver babies in hygienic circumstances but also to counsel mothers about stresses and provide effective psychological interventions. Another example of strengthening health-care systems is to ensure that while district health managers are sourcing antiretrovirals for people with HIV/AIDS, effective treatments for depression are also being made available for those who need them.

A number of mental health indicators can be developed and used to monitor the mental health of target populations, ranging from individual-level indicators—such as rates of depression measured using simple, short questionnaires—to population-level indicators—such as suicide rates and alcohol-related mortality. These prescriptions do not translate into the need for substantial additional resources. In many instances, it is only a broader orientation that is required. Where new resources are needed, they are likely to be cheap and cost-effective.

But perhaps most important of all is to advocate effectively to challenge the nihilism of global health planners, regarding the role of mental health. The acknowledgment of the importance of maternal depression to maternal and newborn health in this year's World Health Report is a welcome step in this direction [33]. Undoubtedly, stigma plays a key role in explaining the lack of acceptance of mental health as a legitimate health concern of people in developing countries. We must challenge this both through research evidence and through ensuring more opportunity for local voices from developing countries to acknowledge their needs and agendas. Above all, the global mental health advocacy discourse needs to reinforce its key message: there is no health without mental health [34].

## Abbreviations

DALY: disability-adjusted life-year
MDG: Millennium Development Goal
YLD: year lived with disability
